# Counterfeit fifty Ringgit Malaysian banknotes authentication using novel graph-based chemometrics method

**DOI:** 10.1038/s41598-022-08821-w

**Published:** 2022-03-22

**Authors:** Nurfarhana Hassan, Tahir Ahmad, Naji Arafat Mahat, Hasmerya Maarof, Foo Keat How

**Affiliations:** 1 Department of Computer and Mathematical Sciences, Universiti Teknologi MARA Cawangan Pulau Pinang, Kampus Permatang Pauh, 13500 Permatang Pauh, Pulau Pinang Malaysia; 2 Department of Mathematical Sciences, Faculty of Science, Universiti Teknologi Malaysia, 81310 Skudai, Johor Malaysia; 3 Department of Chemistry, Faculty of Science, Universiti Teknologi Malaysia, 81310 Skudai, Malaysia; 4 Centre for Sustainable Nanomaterials, Ibnu Sina Institute for Scientific and Industrial Research, Universiti Teknologi Malaysia, 81310 Skudai, Malaysia; 5 Crime Investigation Department, Sentul District Police Headquarters, Royal Malaysia Police, Kuala Lumpur, Malaysia

**Keywords:** Infrared spectroscopy, Cheminformatics, Mathematics and computing, Scientific data

## Abstract

Counterfeiting, in particular, the forgery of banknotes is a serious crime problem and has become a great challenge to the global economies. The forensic science experts have been utilizing chemical technique such as infrared spectroscopy to analyze genuine and counterfeit banknotes. Nevertheless, chemometrics techniques are required to further discriminate the banknotes. In this paper, an advanced fuzzy graph chemometrics method is used to discriminate genuine and counterfeit fifty Ringgit Malaysian (RM50) banknotes. The development of the technique, namely chemometrics fuzzy autocatalytic set (c-FACS) is presented in this paper, together with the results and its comparison to principal component analysis (PCA) method. The results from the c-FACS analysis showed distinct patterns and features of the counterfeit banknotes in the c-FACS plot. Furthermore, the new method is faster than PCA in authentication analysis of counterfeit banknotes. Hence, the c-FACS provides better performance in terms of computing time as compared to PCA, and has the potential in assisting the investigation involving counterfeit banknotes.

## Introduction

Money is a mechanism to facilitate trading of wealth indirectly; not directly as with barter. It may take a physical form as in coins and notes, or may exist as a written (cheque) or electronic account. However, its invention took place even before beginning of written history^1^.

**Figure 1:**
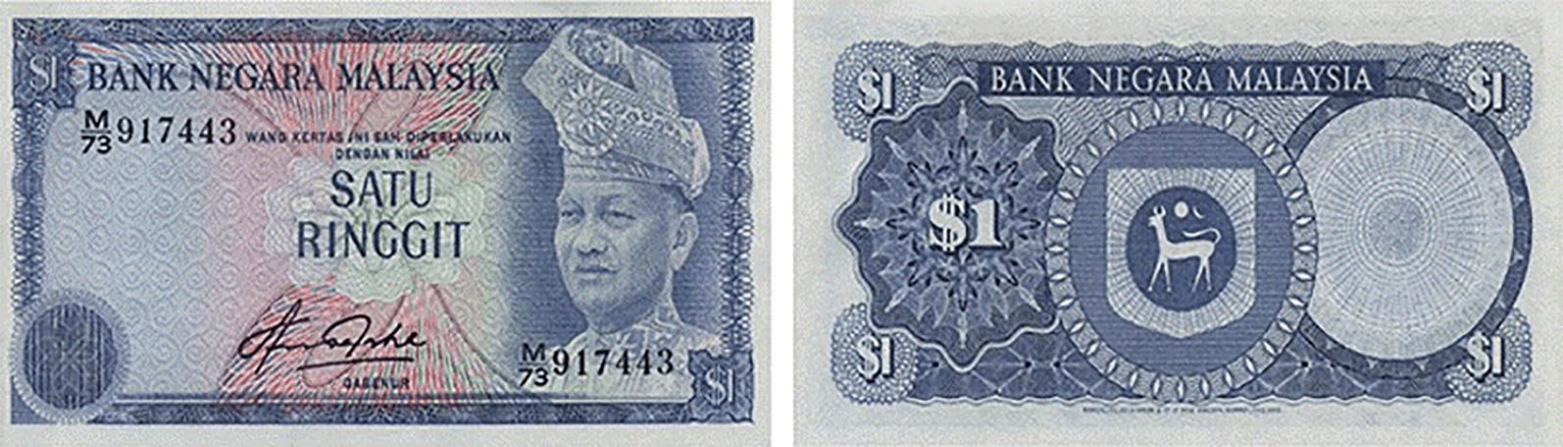
Malaysian $1.

Banknote or paper money was first used in China in the seventh century, and it is believed to be actually developed and appeared in the eleventh century, during the Song dynasty^2,3^. In Europe, the concept of banknotes was first introduced during the thirteenth century and firstly appeared in Sweden in 1661. Bank Negara of Malaysia (BNM) began issuing Malaysian currency notes in June 1967 in five denominations, namely, $1 (see Fig. 1), $5, $10, $50 and $100^4^.

Since then, BNM has added and improved features of Malaysian banknotes with an aim to combat counterfeit money; i.e. anti-counterfeiting measures. Counterfeiting, the forgery of banknotes is a great challenge indeed. It has led to the development of security printing and discrimination methods for genuine and counterfeit banknotes, respectively.

One of the important aspects in a commercial crime investigation is to develop forensic intelligence to differentiate the varying qualities and types of such counterfeited banknotes, necessitating utilization of chemometrics methods for revealing the modus operandi. This may prove useful to streamline the logistic support for narrowing down the search for the perpetrators^5^. Chemometrics is a technique that utilizes mathematical tools to analyze large and complex dataset^6^. It has been emergently used in many areas and applications, in particular, chemical and forensic science, that require rigorous analysis on their dataset^7^. Forensic investigations usually involve with analysis on dataset of collected evidences and crime related substances, such as questioned documents, forgery products and liquid samples^8,9^. The evidences are analyzed using chemical techniques, to obtain the chemical dataset, and further analyzed using chemometrics method to obtain unambiguous and acceptable results. The most common chemical and chemometrics technique used, particularly for authentication of products, are spectroscopy and statistical approach, respectively^10,11^.

In this paper, a chemometrics method using fuzzy graph approach is used for discrimination analysis of fifty Ringgit Malaysia (RM50) Malaysian banknotes. According to the Commercial Crime Investigation Department of the Royal Malaysia Police, RM50 banknotes are the most common Malaysian currency being counterfeited, justifying its choice in this present research (Royal Malaysia Police, personal communication, November 30, 2018). Fourier transform infrared (FTIR) spectroscopy^12,13^ and statistical techniques such as principal component analysis (PCA)^5,14^, partial least square discriminant analysis (PLS-DA)^10,15^ and linear discriminant analysis (LDA)^16^ are commonly used to authenticate banknotes. However, authentication analysis of counterfeit banknotes using fuzzy graph has never been reported in literature. Therefore, in this study, a fuzzy graph method named chemometrics fuzzy autocatalytic set (c-FACS) is introduced and integrated with Fourier transform infrared (FTIR) spectroscopy to distinguish the spectra of RM50 Malaysian banknotes. The coordinated FACS is then further executed to visualize and identify the patterns of the spectra.

## Methods

### Graph and autocatalytic set (ACS)

Graphs are defined as networks of points or nodes that are connected by links. They are described as a set of lines that connect to a set of points. The formal definition of graph is given by Balakrishnan and Ranganathan^17^ as follows:

#### **Definition 1**

A directed graph $G = (V,E)$ is defined by a set of $V$ “vertices” also known as “nodes” and a set $E$ of “edges” or “links” where each edge is an ordered pair of vertices.

**Figure 2:**
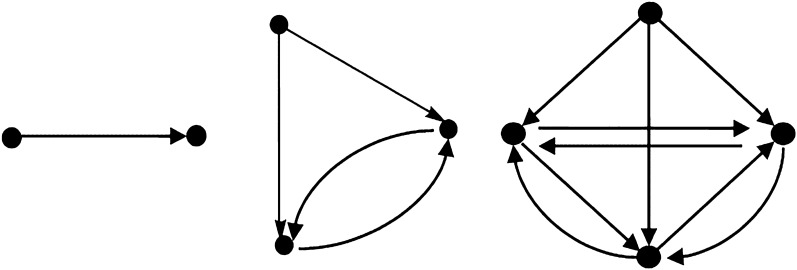
Examples of graphs.

All the graphs in Fig. 2 are directed graphs. A set of vertices or nodes can be represented as $V = \{ v_{1} ,v_{2} ,v_{3} , \ldots ,v_{n} \}$ and $E = \{ e_{1} ,e_{2} ,e_{3} , \ldots ,e_{m} \}$ where the vertices and edges are also known as nodes and links, respectively.

The adjacency matrix of a graph is presented as $C = (c_{ij} )$. Harary^18^ described the adjacency matrix in the following definition.

#### **Definition 2**

Adjacency matrix of graph $G = (V,E)$ with n vertices is a $n \times n$ matrix, denoted by $C = (c_{ij} )$, where $c_{ij} = 1$ if *E* contains a directed link $(j,i)$ (arrow pointing from vertices $j$ to vertices $i$) and $c_{ij} = 0$ otherwise.

**Figure 3:**
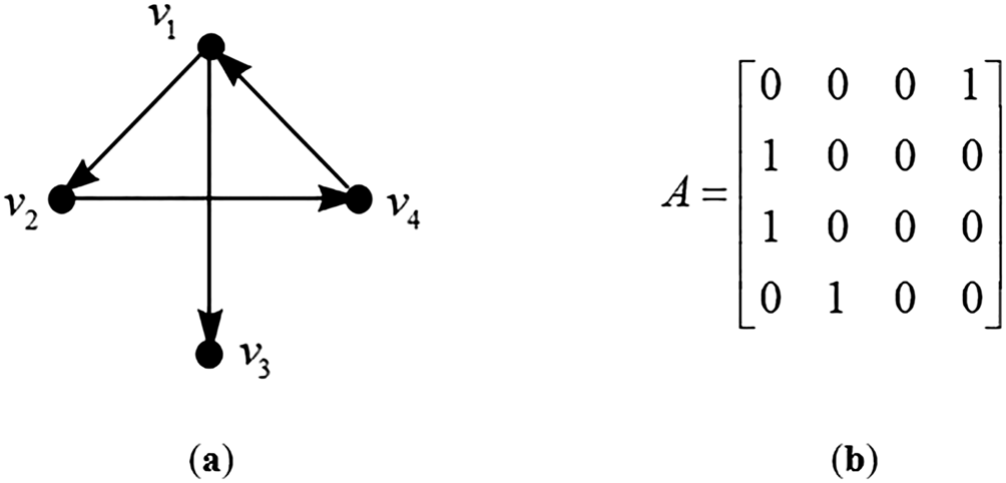
(**a**) A directed graph and (**b**) its adjacency matrix.

The adjacency matrix of graph $G$ can be described as follows:

1 $$ c_{ij} = \left\{ \begin{aligned} &1{\text{ if (}}i,j{)} \in E \\ & 0{\text{ if (}}i,j{)} \notin E \\ \end{aligned} \right. $$

and the entries for matrix $G$ represent the connection between the $i$th and $j$th vertices. Figure 3 shows an example of graph $G$ with four vertices and its adjacency matrix.

The definition of a graph given by Jain and Krishna^19^ is slightly different than Definition 1 above. The $c_{ij} = 1$ if and only if there is a link from node $i$ to node $j$. Basically, their resultant adjacency matrix is the transpose of the adjacency matrix described by Definition 2.

In general, an autocatalytic set is defined as a set of entities or a collection of entities where the word entities can be anything such as people, molecules, or objects. Jain and Krishna^19^ formalized the definition of an autocatalytic set in the form of a graph as follows:

#### **Definition 3**

An autocatalytic set is a subgraph, each of whose nodes has at least one incoming link from a node belonging to the same subgraph.

**Figure 4:**
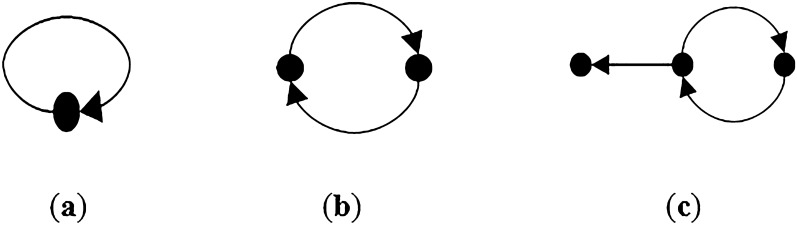
(**a**) A 1-cycle and ACS, (**b**) A 2-cycle and ACS and (**c**) an ACS but not irreducible.

The concept of an autocatalytic set for a graph is defined as vertex $j$ catalyst vertex $i$. The simplest of ACS graph is a vertex with 1-cycle. Figure 4 illustrates some samples of autocatalytic sets.

### Chemometrics fuzzy autocatalytic set (c-FACS)

**Figure 5:**
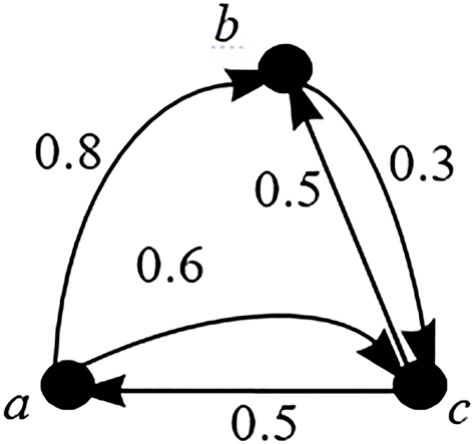
An FACS.

In 2010, Ahmad et al.^20^ introduced the concept of fuzziness into ACS. They formed a new concept called fuzzy autocatalytic set (FACS) to model a waste incineration process, whereby the edges of the graph have fuzzy membership values. The formal definition of FACS by Ahmad et al.^20^ is illustrated in Fig. 5 and defined formally as follows:

#### **Definition 4**

Fuzzy autocatalytic set (FACS) is a sub graph each of whose nodes has at least one incoming link with membership value $\mu (e_{i} ) \in \left( {0,1} \right], \, \forall e_{i} \in E$.

Later in 2020, Hassan et al.^21^ adapted the FACS method in combination with a chemical technique, namely Fourier transform infrared (FTIR) spectroscopy, to analyze chemical dataset of gelatin. The new advanced method is called chemometrics fuzzy autocatalytic set (c-FACS). Since then, the c-FACS method has been established and utilized in other various applications involving food authentication^22^, pattern and classification analysis for COVID-19 outbreak in Malaysia^23^. Their results also coincide with the results of principal component analysis (PCA), and thus verified the accuracy and effectiveness of the c-FACS method. Furthermore, the performance of the c-FACS in the respective researches were also compared to PCA, and c-FACS showed faster computing time. Thus, the c-FACS method has been proven to be effective in authentication analysis, in particular, to identify and differentiate patterns of dataset.

**Figure 6:**
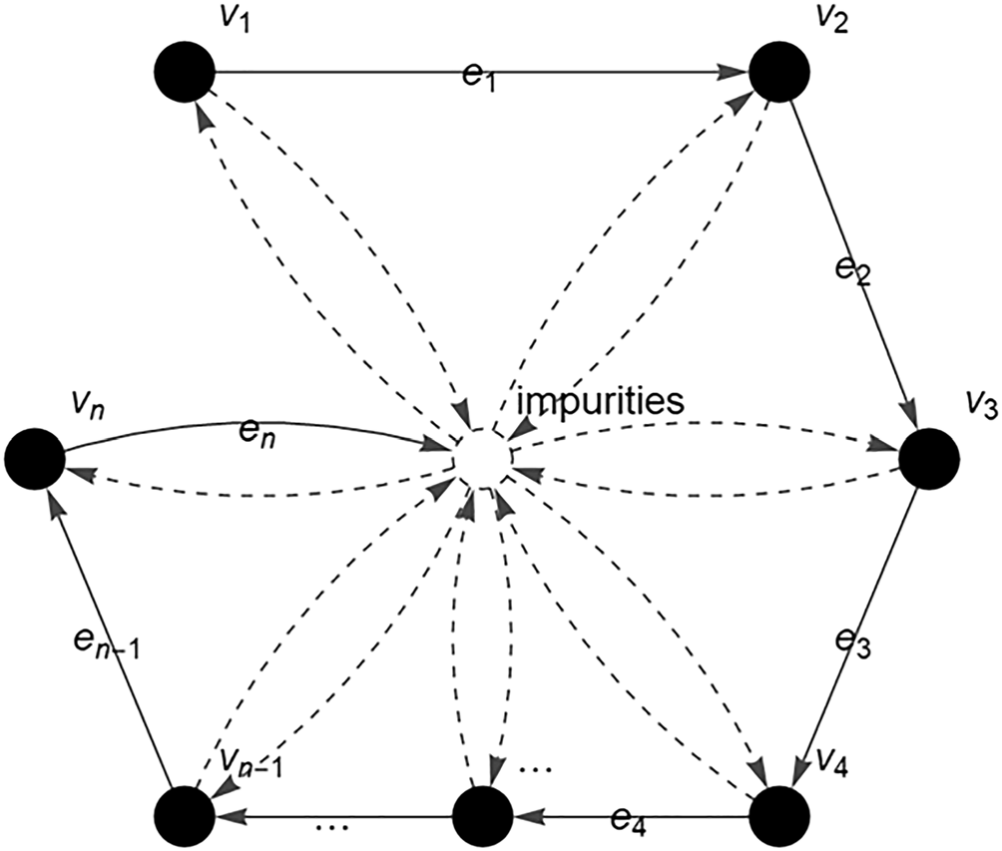
Fuzzy autocatalytic set (FACS) graph of FTIR banknote’s spectra.

The c-FACS involves the representation of the FTIR spectra in the form of a FACS graph and its adjacency matrix for further analysis to identify the dominant output. In this paper, firstly, the FTIR spectra of the banknotes are obtained. Then, the process of infrared absorption by the banknote molecule during FTIR analysis is represented in the form of vertices and edges. Each vertex in $V = \{ v_{1} ,v_{2} ,v_{3} ,v_{4} , \ldots ,v_{n - 1} ,v_{n} \}$ represents the wavenumber (cm^−1^) and an edge in $E = \{ e_{1} ,e_{2} ,e_{3} ,e_{4} , \ldots ,e_{n - 1} ,e_{n} \}$ is the transitional path of a given molecule throughout the FTIR analysis (see Fig. 6).

In short, the FACS graph describes the flow process and movement of a molecule from one wavenumber of light to the next wavenumber during the FTIR analysis. The detected amount of infrared radiation absorbed by the molecule at each wavenumber (cm^−1^) is represented as the absorbance value (A) and plotted in the FTIR spectrum^24^. In addition, the presence of impurities during the analysis is also considered in the FACS graph. The obtained graph is then converted into a matrix for further analysis. The entries of the matrix are the membership values, $\mu \left( {v_{i} } \right)$ of the absorbance readings $(Q_{i} )$ at each wavenumber. The $\mu \left( {v_{i} } \right)$ is called fuzzy absorbance value and defined by:

2 $$ \mu \left( {v_{i} } \right) = \left\{ \begin{aligned} & 0 \quad \quad \quad \quad\quad\quad{\text{ if }}Q_{i} < 0 \hfill \\ & 1 \quad \quad\quad\quad\quad\quad{\text{ if max(}}Q_{i} ){\text{ for }}i = 1, 2, 3, \ldots , n \hfill \\ & {{Q_{i} } \mathord{\left/ {\vphantom {{Q_{i} } {\max (Q_{i} )}}} \right. \kern-\nulldelimiterspace} {\max (Q_{i} )}} \quad {\text{ others}} \hfill \\ \end{aligned} \right. $$

Hassan et al.^21^ constructed the c-FACS algorithm. The algorithm is then coded and named ‘Multisystem Dynamic Identification of Gelatin Sources © 2020 Universiti Teknologi Malaysia—All Rights Reserved’. It is outlined as follows.
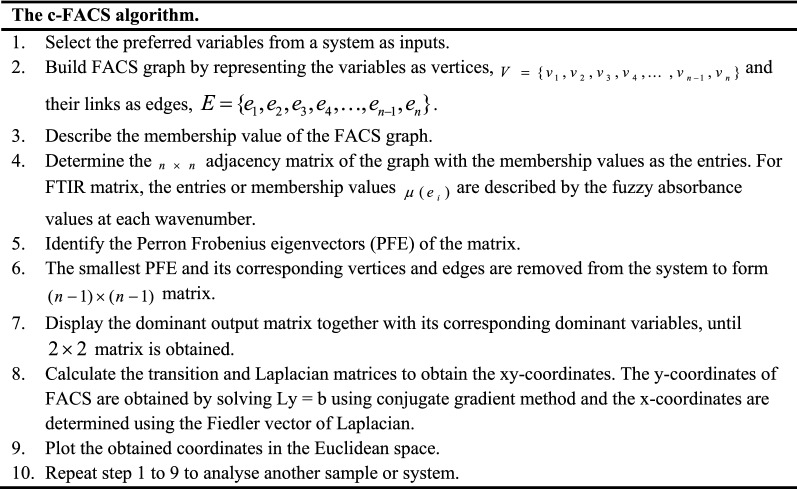


This algorithm is then executed on authentication of RM50 banknotes. The implementation of the procedure on RM50 banknote samples is described in the following sections.

## Implementation

### Samples

This present study used the 28 pieces of counterfeit RM50 banknotes (fourth series) introduced into circulation since July 2009 provided by Commercial Crime Investigation Department (CCID) of Royal Malaysia Police, and 8 pieces of genuine RM50 banknotes withdrawn from auto teller machines (ATMs) in Universiti Teknologi Malaysia (UTM), Taman Sri Pulai and Taman Universiti, Skudai, Johor Bahru as standards. Methanol (95% purity) was used for cleaning the metal stage of the ATR-FTIR after each run. The banknotes samples were observed according general visual and physical examinations for its security features under Video Spectral Comparator (VSC40HD) as described by BNM for its authenticity.

### Instrument and data acquisition

**Figure 7:**
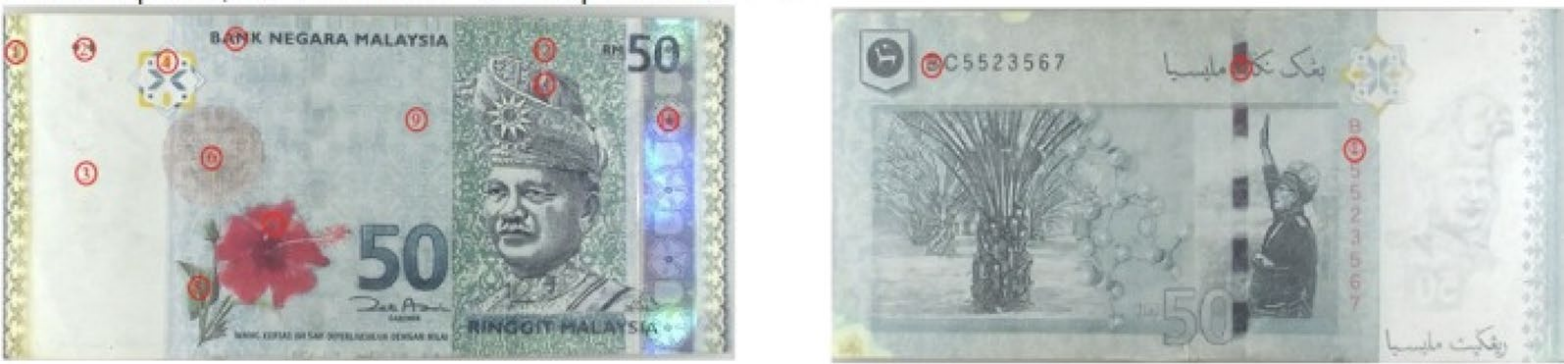
The RM 50’s marked areas.

Both the genuine and counterfeit RM50 banknotes were observed under VSC40HD (Foster and Freeman, UK) to visualize its security feature by adjusting the magnification power (2.05× to 16×) and using different wavelength of UV light (254 nm UV, 312 nm UV and 356 nm UV). Perkin-Elmer ATR-FTIR Spectrometer Frontier (Waltham, U.S.A.) coupled with deuterated triglycine sulfate (DTGS) detector was used to differentiate the chemical components used on the genuine and counterfeit banknotes in the fingerprint region range 1800–650 cm^−1^. This fingerprint region has been suggested as substantially useful for differentiating the genuine and counterfeit products including the Malaysian Ringgit 100 currency^5,11^, indicating the possibility to chemically differentiate the two categories. Triplicate scans were performed on five areas from different section in the obverse and reverse side of the banknote samples. The selected five areas were the watermark portrait (O1), multicolour latent image (O2), the predominant colour of green–blue areas (O3 and O4) on the obverse and the serial number (R1) on reverse of the RM50 banknotes, respectively. The area of holographic stripe and security thread of the RM50 banknotes were not taken into study due to the holographic stripe and security thread were pasted on some of the counterfeit RM50 banknotes instead of embedded into the genuine RM50 banknotes during the production. The holographic stripe on the obverse and the security thread on the reverse of the RM50 banknotes were excluded in this study for a relatively impartial comparison reason (see Fig. 7). A total of 15 cumulative scans were taken for each sample with the resolution of 4 cm^−1^ in the spectral fingerprint range. Base line correction followed by smoothing of the spectrum were done prior to saving the spectrum. The spectrum data was imported and tabulated to Microsoft Excel format for further analysis. An average of the three spectra was employed throughout study.

## Results and discussion

### Comparison of ATR-FTIR spectrum of the obverse area between genuine and counterfeit RM50 banknotes

**Figure 8:**
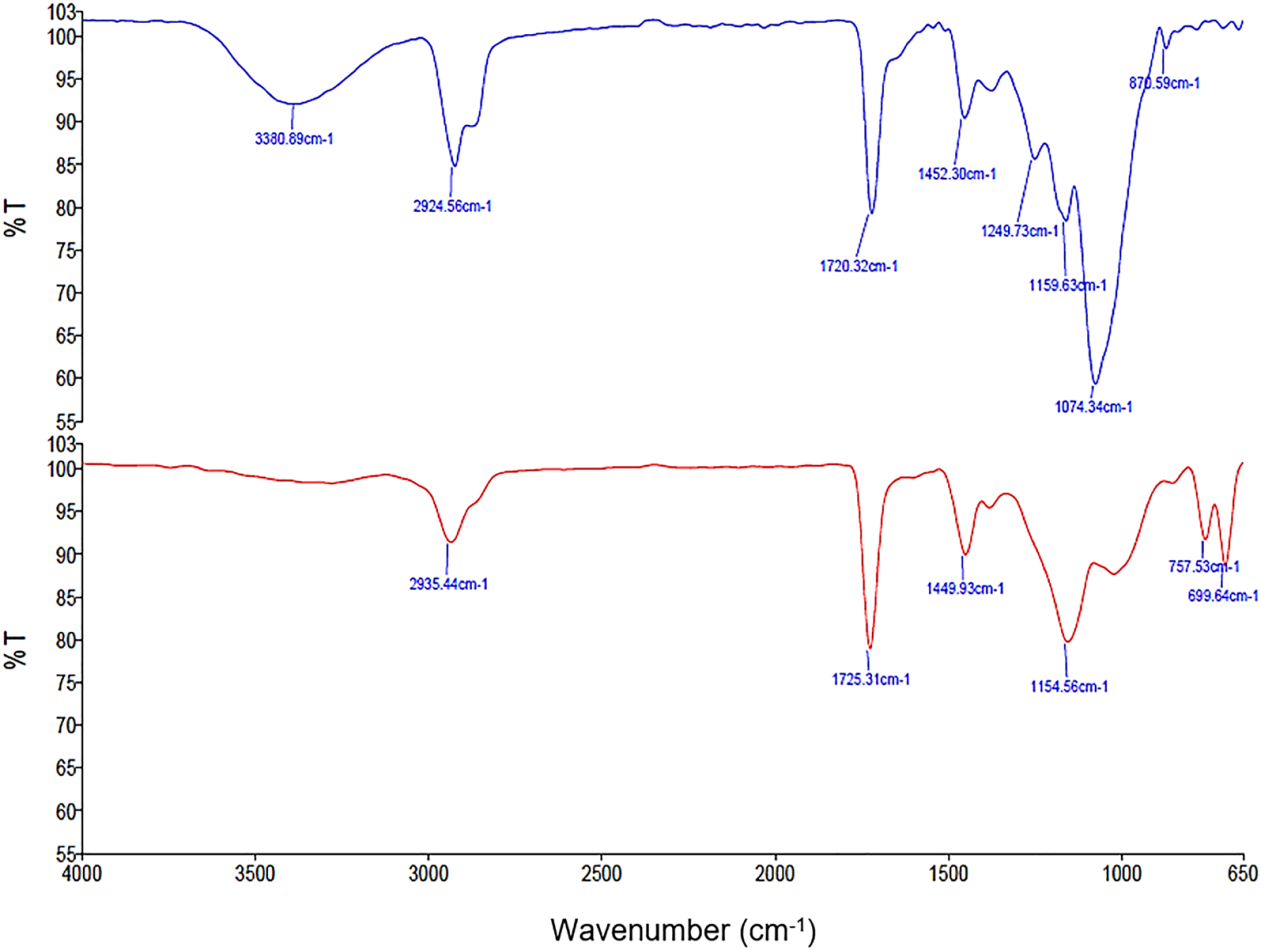
ATR-FTIR spectra comparison of the obverse area of genuine (blue spectrum) and counterfeit (red spectrum) RM50 banknotes.

Figure 8 shows the spectrum of area on the obverse (O1) of a genuine and counterfeit RM50 banknotes. Similar with that reported by previous researchers^5^, the fingerprint region (1800–650 cm^−1^) provided greater unique characteristics that may prove useful for discriminating the genuine and counterfeit samples than that of 3400–650 cm^−1^ region. Only slight variations existed between the genuine and counterfeit banknotes within the 3400–650 cm^−1^ region, whereby the carboxylic group (OH) observed at 3380 cm^−1^ for the genuine banknotes was not observed in the counterfeit ones. As for the C–H stretching at wavenumbers 2924–2935 cm^−1^, the functional group prevailed for both the genuine and counterfeit banknotes analysed here.

The genuine and counterfeit RM50 banknotes show three similar peaks and intensity at around 1720 cm^−1^, 1450 cm^−1^ and 1160 cm^−1^. The peaks at 1726 cm^−1^ and 1450 cm^−1^ can be attributed to carbonyl group of calcium carbonate (CaCO_3_) which was used as an additive in the manufacture of paper^12^. The peak at around 1160 cm^−1^ was due to C–O groups stretching vibrations of cellulose substrate which is the backbone of the banknotes^13^. The intense peak at 1075 cm^−1^ is distinctive of cellulose-based materials, due to C–O groups stretching vibrations, which was not appeared or in less intensity in the counterfeit RM50 banknotes, can be used to discriminate between the counterfeit and the genuine RM50 banknotes. The intensity of the cellulose peaks is influenced by the cotton composition of the paper^13^.

There are also differences in the region of 800–650 cm^−1^ with the counterfeit banknotes showing more peaks which could be resulted from the carbonate peaks originated from the presence of calcium carbonate as additives^12^. Thus, the genuine and counterfeit banknotes can clearly be differentiated by studying the IR spectra of this region. From the obtained IR results, it can be observed that the discrimination between genuine and counterfeit RM50 banknotes were based on the comparison between paper compositions materials but not on printing ink components. This could due to the printing ink used to produce the RM50 banknotes were made of inorganic pigments, thus they do not absorb in mid-IR region (4000–650 cm^−1^) but only shows their characteristic absorption bands in low wave IR region (500–230 cm^−1^)^13,25^.

### Comparison of ATR-FTIR spectrum of the reverse area between genuine and counterfeit RM50 banknotes

**Figure 9:**
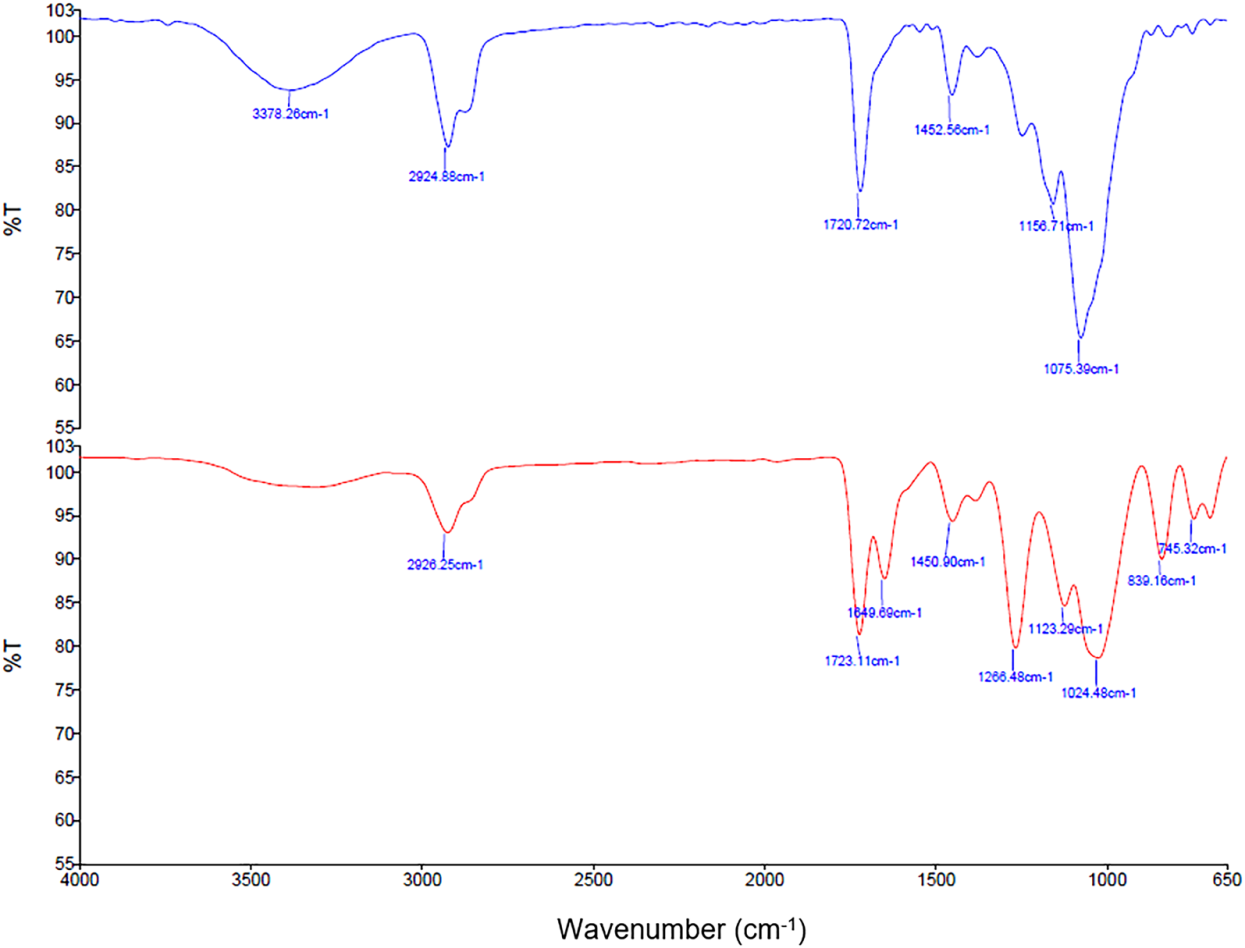
ATR-FTIR spectra comparison of the reverse area of genuine (blue spectrum) and counterfeit (red spectrum) RM50 banknotes.

The spectra of reverse area (excluding security thread at area) of genuine and counterfeit RM50 banknotes are shown in Fig. 9. The spectrum on the reverse area of genuine RM50 banknotes shows an identical composition, regardless of the position to the obverse areas of the genuine RM50 banknotes. The differences between the spectra between genuine and counterfeit RM50 banknotes can be observed fundamentally in the presence of the extra peaks at 1300–1000 cm^−1^ and 850–700 cm^−1^. The peaks at 1300–1000 cm^−1^ on the reverse of counterfeit RM50 banknotes are typical C–O groups stretching of cellulose bands, which could be resulted from the paper composition materials used in producing the counterfeit RM50 banknotes. On top of that, the peaks appeared in the region of 850–700 cm^−1^ are likely resulted from the presence of calcium carbonate, which is often used as the filler during the paper production^12,13^. As for the wavenumbers outside the fingerprint region (3400–650 cm^−1^), the carboxylic group (OH) observed at 3378 cm^−1^ for the genuine banknotes was not found in the counterfeit samples. Nonetheless, the C–H stretching at wavenumbers 2924–2926 cm^−1^ remained identifiable for both the genuine and counterfeit samples.

### Chemometrics fuzzy autocatalytic set (c-FACS) analysis

**Figure 10:**
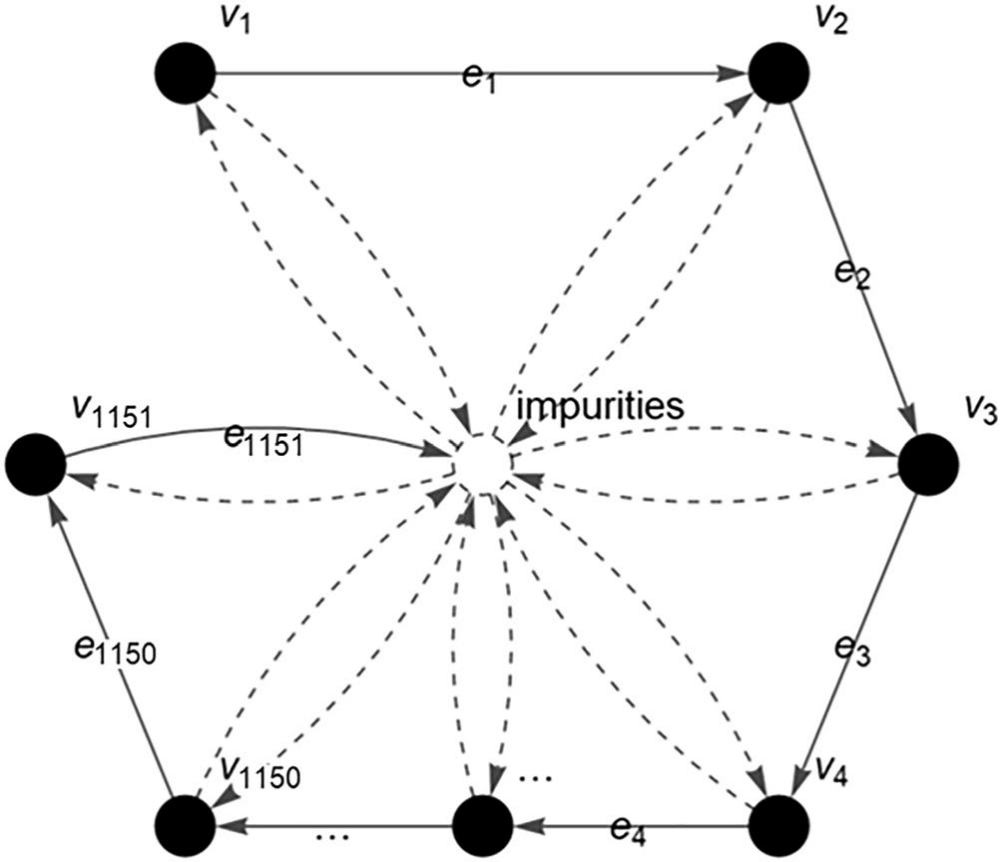
The FACS graph of RM50 banknotes’ spectra at wavenumbers of 1800–650 cm^−1^.

The c-FACS is implemented on the genuine and counterfeit RM50 banknotes spectra obtained from Section above. The banknotes spectra are analysed using the c-FACS algorithm to identify their patterns and signature differences. The spectra of the samples at wavenumbers of region 1800–650 cm^−1^ are represented in a form of FACS graph (see Fig. 10). The set of vertices, $V = \{ v_{1} ,v_{2} ,v_{3} ,v_{4} , \ldots ,v_{1150} ,v_{1151} \}$ represents the wavenumbers position, while the set of edges, $E = \{ e_{1} ,e_{2} ,e_{3} ,e_{4} , \ldots ,e_{1150} ,e_{1151} \}$ represents the transition to the next wavenumbers.

**Figure 11:**
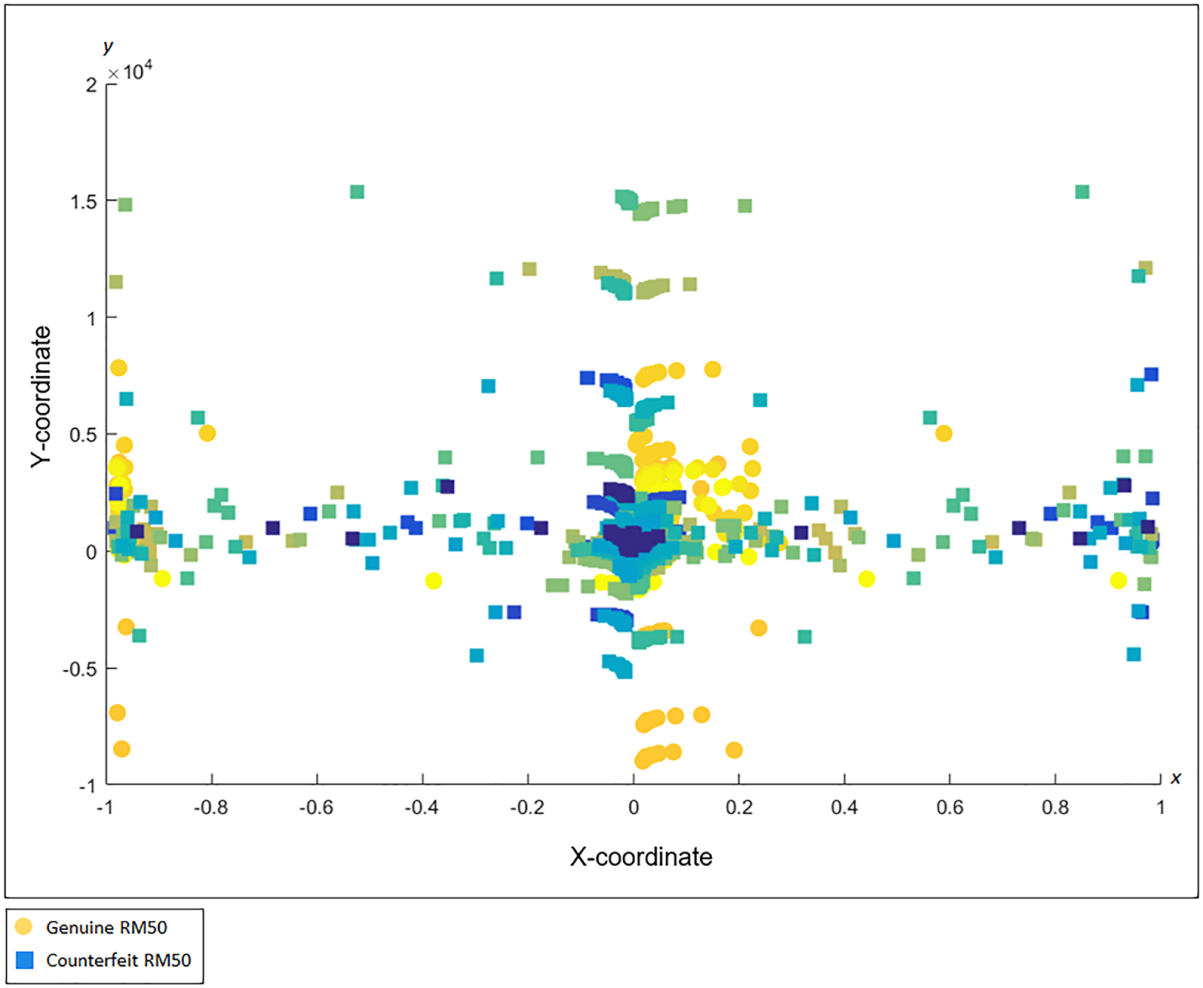
Coordinated FACS of Genuine and Counterfeit RM50. This figure was generated using MATLAB version R2016a (Mathworks, Natick, MA; https://www.mathworks.com).

The c-FACS algorithm is performed using MATLAB software version R2016a (Mathworks, Natick, MA; https://www.mathworks.com) to obtain and generate figures of the coordinated FACS patterns of the genuine and counterfeit RM50 samples. In the algorithm, the vertices in Fig. 10, which represent the wavenumbers, are positioned and plotted in Euclidean space with respect to their respective coordinates. The coordinates are obtained through computations on Laplacian matrix in the algorithm. In short, the c-FACS algorithm converts the graph in Fig. 10, into its coordinated form. As a result, some patterns of the graph can be observed. Each node or vertex that creates the patterns in the graph, represent wavenumbers. Thus, the patterns of the nodes, or particularly wavenumbers are the significant variables that are used to differentiate the samples. Figure 11 yields the output of the c-FACS for the RM50 banknotes. The different colour of nodes indicate the different sources of the samples. The nodes for genuine RM50 banknotes are plotted in yellowish circles, while the nodes for counterfeit samples are in bluish squares.

The coordinated FACS result shows distinct patterns for the genuine and counterfeit samples at certain areas. In particular, the coordinated patterns for counterfeit samples are observed to be dominantly clustered at the area of x-axis from − 0.2 to − 0.8 and 0.8 to 1, while only few nodes of genuine samples are observed in these areas. The patterns for the genuine samples nodes are mostly overlapped and clustered at x-axis from 0 to 0.2 and at − 1 areas. Additionally, the nodes that are dominantly clustered at the respective x-axis are identified to be the nodes that represent the wavenumbers of 1460–1393 cm^−1^. The results indicated that the nodes of these wavenumbers for genuine and counterfeit samples appear in distinct areas. Hence, their patterns can be used as signature identification of the samples, whereby, for counterfeit banknotes, the nodes of 1460–1393 cm^−1^ are clustered at x-axis from − 0.2 to − 0.8 and 0.8 to 1, while the nodes of 1460–1393 cm^−1^ for genuine samples are clustered at x-axis from 0 to 0.2 and at − 1 areas. Thus, the patterns of the nodes, or particularly the wavenumbers are the significant variable that can be used as signature identification to differentiate the genuine and counterfeit banknotes.

**Figure 12:**
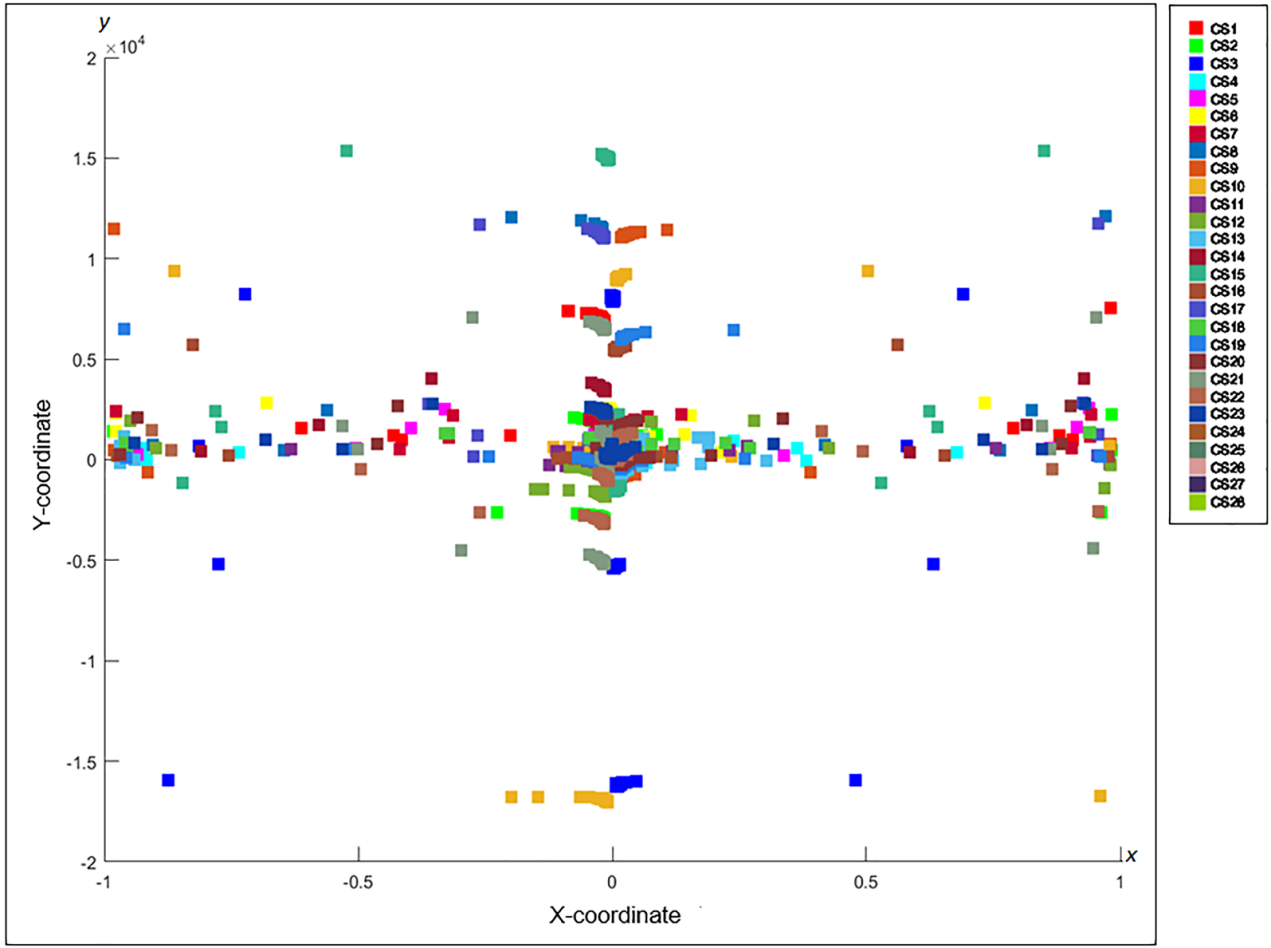
Coordinated FACS of Counterfeit RM50. This figure was generated using MATLAB version R2016a (Mathworks, Natick, MA; https://www.mathworks.com).

In addition, the counterfeit RM50 banknotes are also analysed separately using the c-FACS, to investigate whether they are produced by different manufacturers. The coordinated patterns for the 28 samples of counterfeit RM50 banknotes are shown in Fig. 12.

**Figure 13:**
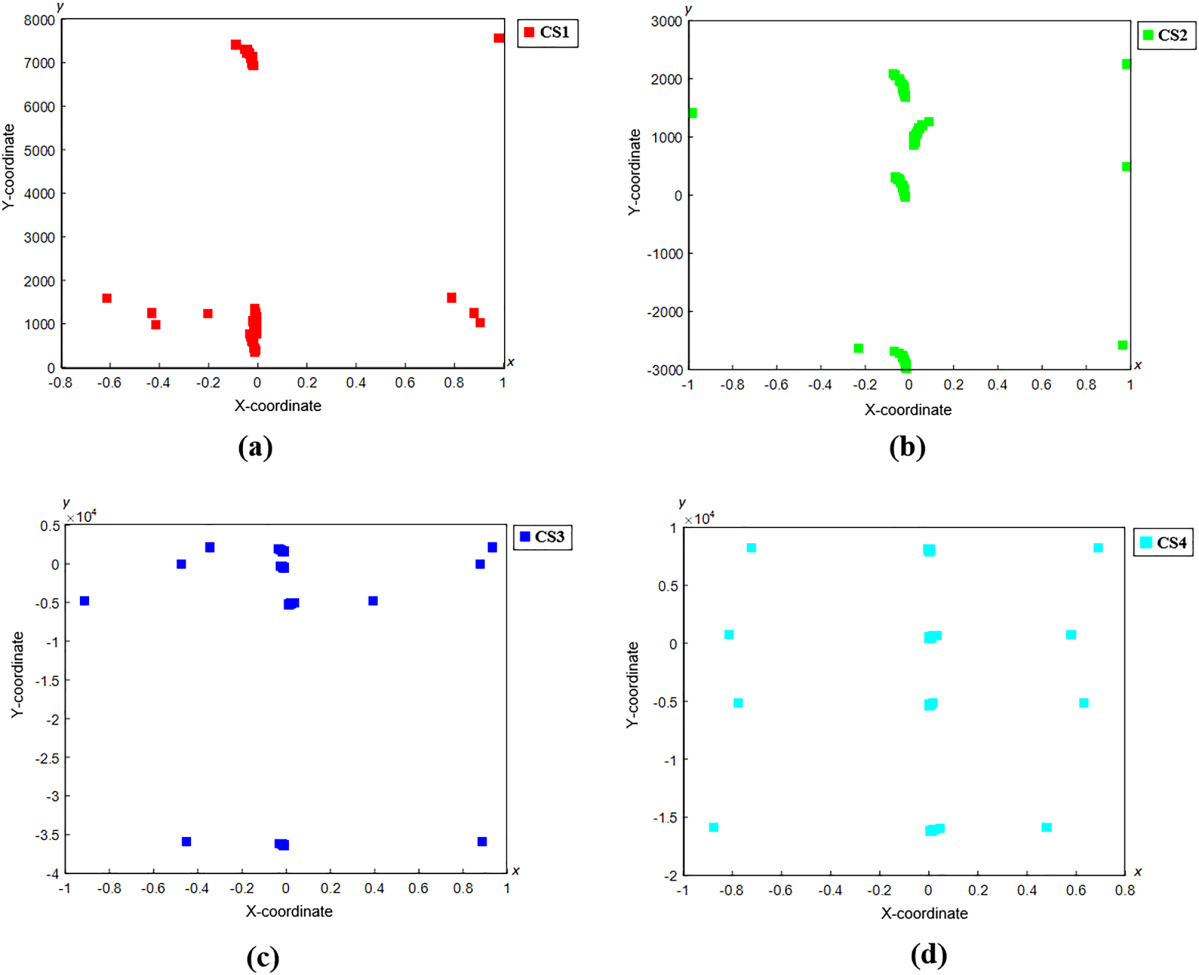
Coordinated FACS of Individual Counterfeit RM50 (**a**) Sample 1 (CS1), (**b**) Sample 2 (CS2), (**c**) Sample 3 (CS3) and (**d**) Sample 4 (CS4). This figure was generated using MATLAB version R2016a (Mathworks, Natick, MA; https://www.mathworks.com).

The coordinated FACS of all 28 samples of RM50 counterfeit banknote nodes are scattered and overlapped, particularly in the middle area of the graph. The RM50 counterfeit samples are hard to be differentiated, due to the possibilities of most of the samples have similar properties and patterns, thus they overlapped each other. Hence, each RM50 counterfeit sample is analysed individually to observe its pattern and compare with the others. The coordinated FACS of counterfeit RM50 sample 1 (CS1), sample 2 (CS2), sample 3 (CS3) and sample 4 (CS4) are shown in Fig. 13, and the coordinated FACS for other remaining samples are displayed in “Supplementary file 1: Appendix A”.

The coordinated FACS patterns for all 28 counterfeit samples (CS) of RM50 are compared. The differences between the patterns are observed, particularly at four areas of x-axis from − 0.8 to − 1, 0 to − 0.2, 0 to 0.2 and 0.8 to 1. The coordinated nodes for CS1, CS2, CS3, CS6, CS8, CS9, CS16, CS17, CS21, CS25 and CS28 are mainly clustered at x-axis from 0 to − 0.2 and 0.8 to 1. The coordinated nodes for CS4, CS5, CS7, CS10, CS15, CS19, CS20, and CS26 are mostly clustered at x-axis from 0 to 0.2 and − 0.8 to − 1. Similarly, nodes for CS11, CS12, CS13, CS14, CS18, CS22, CS23, CS24 and CS27, appear at the same four areas of x-axis. The patterns of the samples at the four areas are summarized in Table 1.

**Table 1:** The clustering of nodes of counterfeit sample (CS) of RM50.

CS	Presence of nodes at x-axis			
	− 0.8 to − 1	0 to − 0.2	0 to 0.2	0.8 to 1
1		–		–
2		–		–
3		–		–
4	–		–	
5	–		–	
6		–		–
7	–		–	
8		–		–
9		–		–
10	–		–	
11	–	–	–	–
12	–	–	–	–
13	–	–	–	–
14	–	–	–	–
15	–		–	
16		–		–
17		–		–
18	–	–	–	–
19	–		–	
20	–		–	
21		–		–
22	–	–	–	–
23	–	–	–	–
24	–	–	–	–
25		–		–
26	–		–	
27	–	–	–	–
28		–		–

The nodes of each sample are observed to be mainly clustered at certain areas. The clusters of nodes at the particular areas could signify certain properties of the counterfeit money. Thus, the samples with similar clusters areas may have similar properties and were possibly manufactured by similar individuals. The c-FACS analysis showed that there is possibility that the counterfeit samples may come from three different manufacturers due to the clustering of the nodes at the observed x-axis areas in Table 1. The CS1, CS2, CS3, CS6, CS8, CS9, CS16, CS17, CS21, CS25 and CS28 have similar clusters areas, thus these samples may come from a similar manufacturer. The CS4, CS5, CS7, CS10, CS15, CS19, CS20, and CS26 may also manufactured by other manufacturer, while CS11, CS12, CS13, CS14, CS18, CS22, CS23, CS24 and CS27 were produced by another manufacturer. Thus, the c-FACS is able to discriminate manufacturers of the counterfeit samples by identified clusters areas. These findings are particularly useful, considering that similar attempts reported by previous researchers, utilizing Partial Linear Square-Discriminant Analysis were unable to separate the different counterfeit RM100 banknotes^13^. This proves that the new method introduced here has better resolution that can be useful for forensic practical caseworks, and therefore should be assessed against other types of counterfeit currencies.

### Principal component analysis (PCA)

Principal component analysis (PCA) is a well-established method for authentication analysis. The PCA is a statistical method that reduces the dimension of the variables and transform them into several principal components (PCs)^26^. The results for the classification of samples are displayed in a form of PCA score plot. The scores for principal component 1 (PC1) and principal component 2 (PC2) are commonly used for the plot since the first two PCSs explain the most variances of the dataset^26,27^.

**Figure 14:**
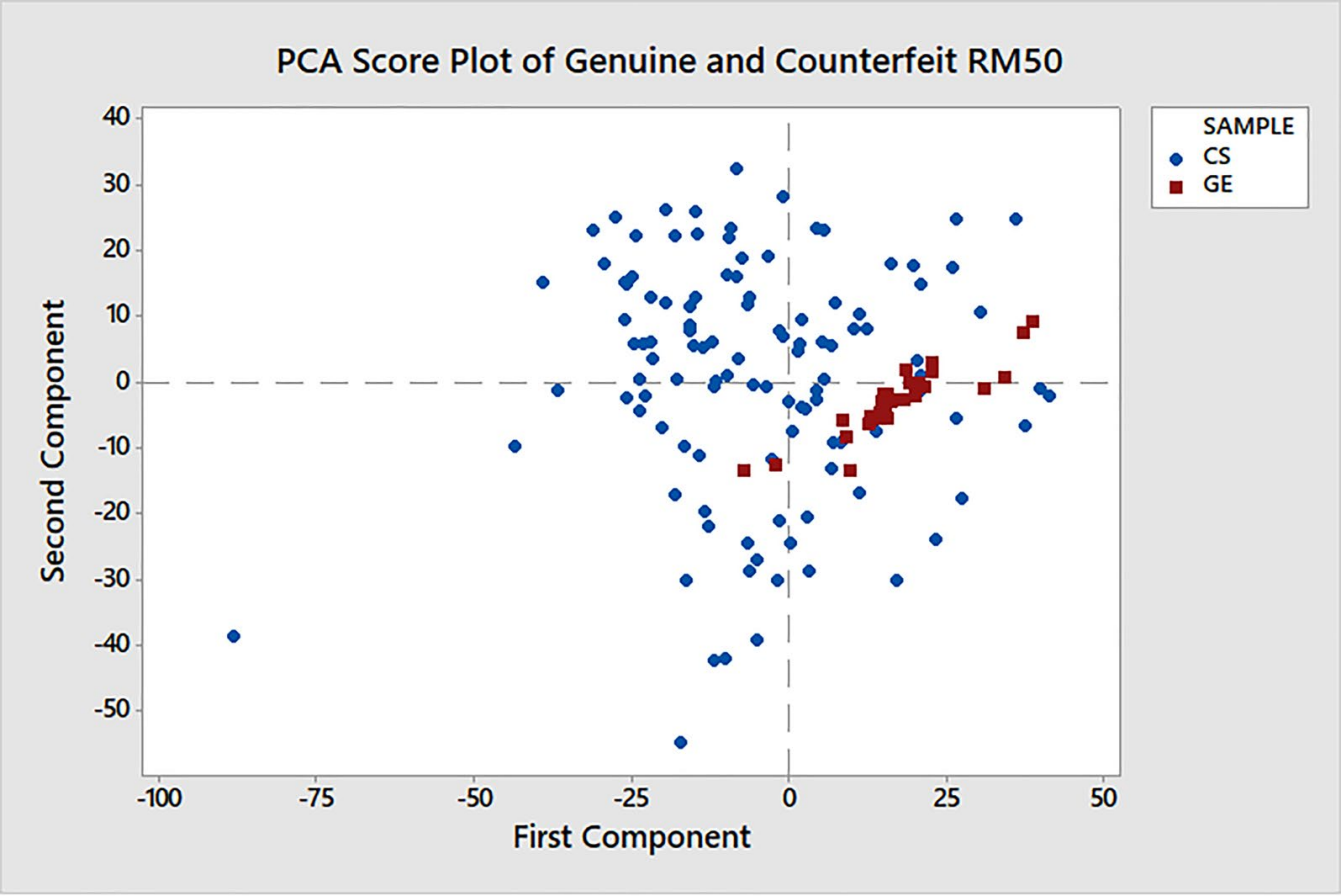
The PCA Score Plot of Genuine (GE) and Counterfeit (CS) RM50 Samples. This figure was generated using Minitab version 17 (Minitab Inc., PA, USA; https://www.minitab.com).

**Figure 15:**
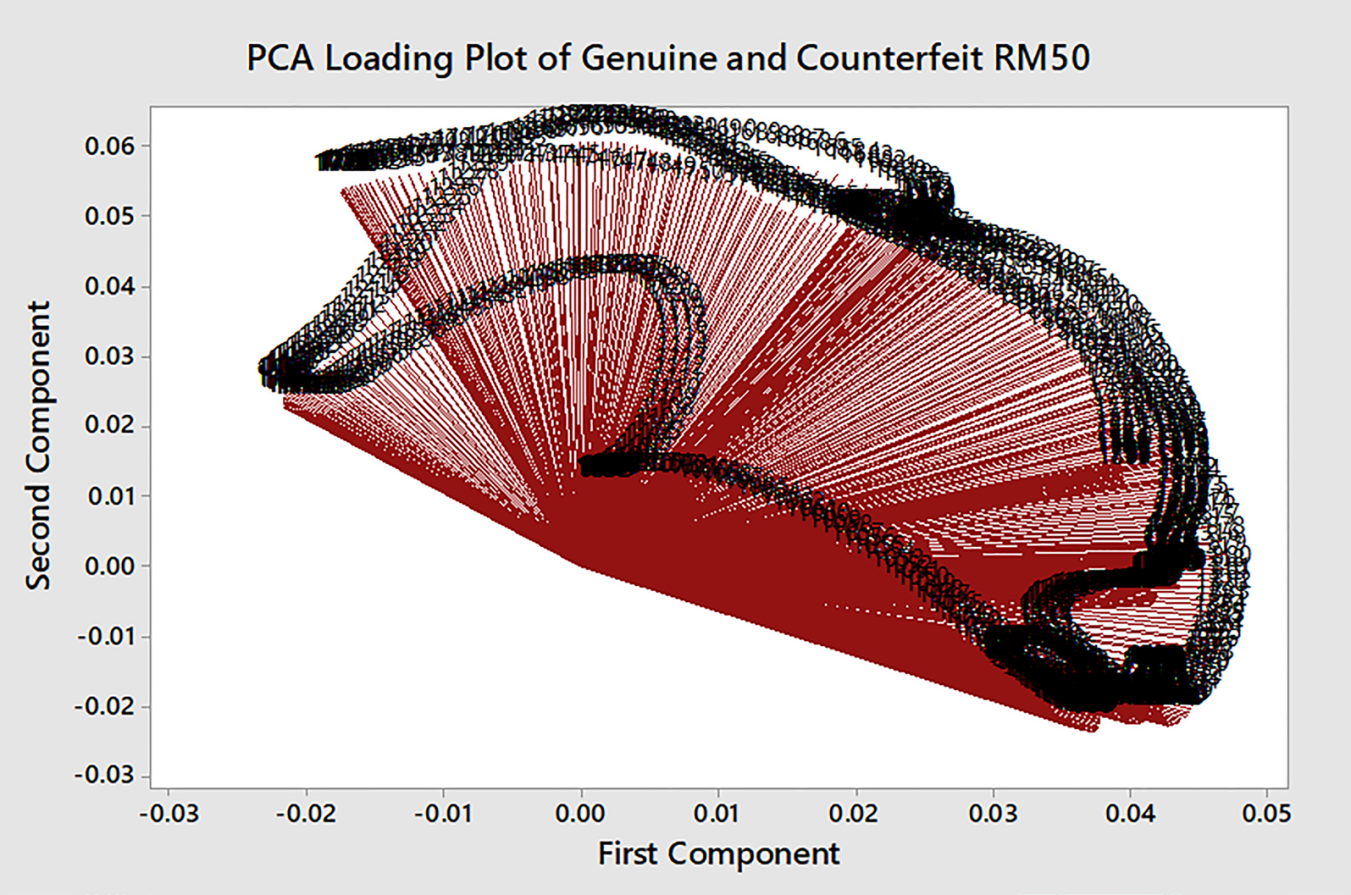
The PCA Loading Plot of Genuine (GE) and Counterfeit (CS) RM50 Samples. This figure was generated using Minitab version 17 (Minitab Inc., PA, USA; https://www.minitab.com).

In this paper, the PCA is used to analyse the genuine and counterfeit RM50 banknote samples and its results are compared to the c-FACS. The PCA is performed using Minitab software version 17 (Minitab Inc., PA, USA; https://www.minitab.com) to generate the PCA plots. The PCA decomposes the data matrix to PCs that describe the variations in the data set. The scores of the PCs represent the coordinates of the observations in the space formed by axes of PCs called score plot. In this analysis, the PC1 and PC2 are used to generate the axes, and the scores of each PC are then plotted. The R2 represents explanatory of variance ability and Q2 shows the predictive ability of the model^28,29^. The PC1 yields R2 = 0.3777 and Q2 = 0.3516. On the other hand, R2 and Q2 for PC2 are 0.6227 and 0.5966 respectively. Eriksson^29^ also pointed out that when Q2 > 0.9, then the predictive performance is excellent and when Q2 > 0.5, it is considered good. Furthermore, the difference between R2 and Q2 must be less than 0.2. Hence, the obtained R2 and Q2 values are considered acceptable since their differences are also small. The results of PCA score and loading plot for genuine and counterfeit money are shown in Figs. 14 and 15.

**Figure 16:**
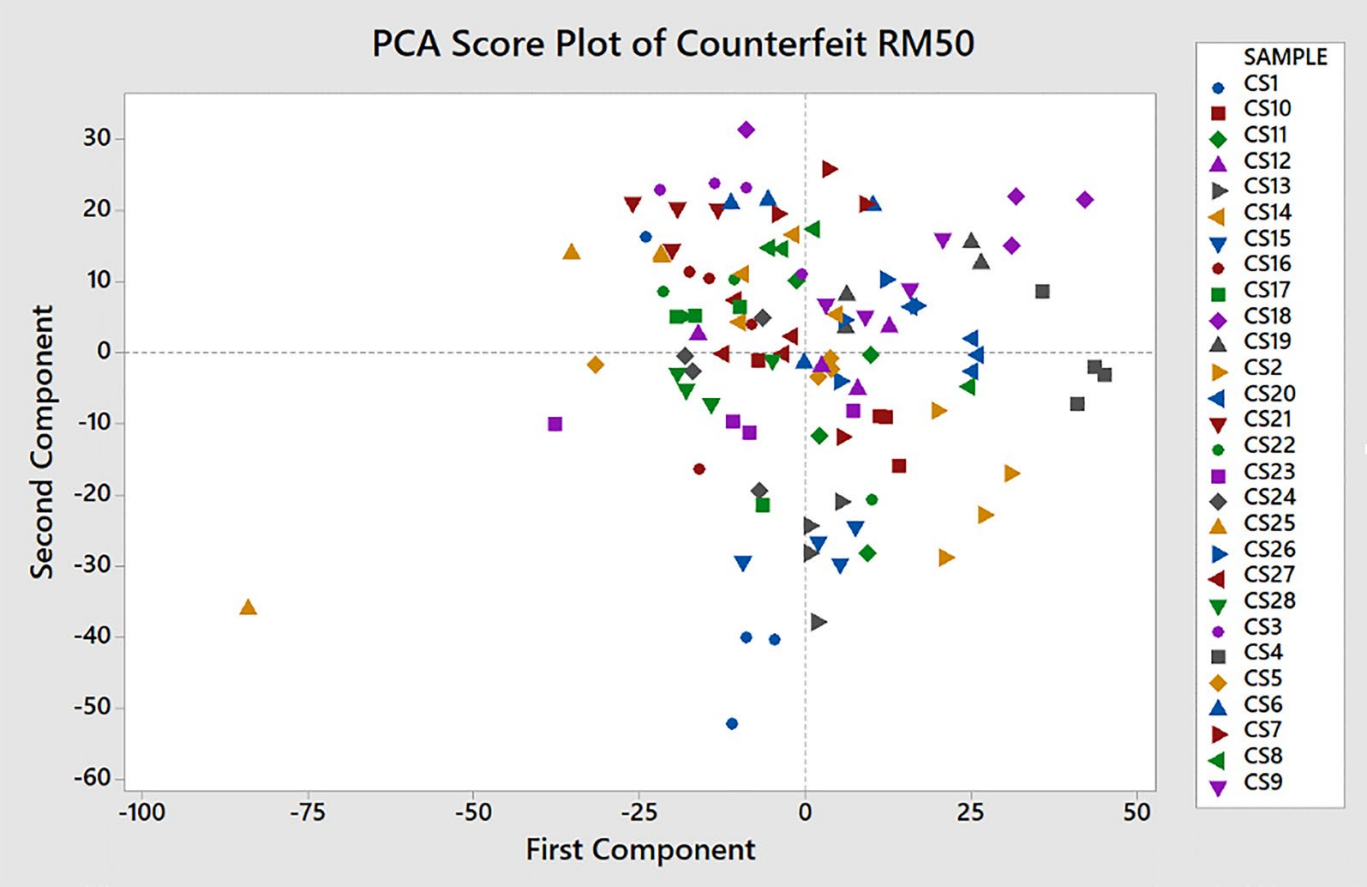
The PCA Score Plot of Counterfeit (CS) RM50 Samples. This figure was generated using Minitab version 17 (Minitab Inc., PA, USA; https://www.minitab.com).

The classifications and clusters of the banknotes samples can be observed in the score plot. The performances of c-FACS and PCA are also compared with respect to their computing time. The c-FACS performed faster, with elapsed time of 56.73 s compared to PCA (74.65 s) for analysis of the genuine and counterfeit RM50 banknotes. The PCA score plot in Fig. 14 shows the patterns of genuine and counterfeit samples are overlapped with each other, while the loading plot in Fig. 15 exhibits unclear and overlapped results and thus the significant wavenumbers that corresponds to the samples are failed to be identified. Furthermore, the nodes of genuine samples in the score plot are observed to be clustered in a particular area. Thus, the PCA showed that the genuine RM50 samples may have distinct characteristics compared to the counterfeit samples, with respect to their clusters. The nodes of the counterfeit samples are dispersed at different areas. Thus, the counterfeit samples are analysed separately to observe their patterns. The results are shown in Fig. 16.

The PCA score plot of the counterfeit RM50 samples show that the nodes of the samples are mostly clustered close to each other. Thus, the classifications of the samples are studied with respect to the axis of the first component (PC1). The samples that are clustered mainly at the right side or positive ranges area of the first component (PC1) are CS2, CS4, CS5, CS7, CS9, CS10, CS11, CS12, CS13, CS15, CS18, CS19, CS20, CS24 and CS26, while the samples that are clustered mainly at the left side or negative ranges of the axis are CS1, CS3, CS6, CS8, CS14, CS16, CS17, CS21, CS22, CS23, CS25, CS27 and CS28. These classifications of the counterfeit samples using PCA are almost similar with the c-FACS analysis, whereby CS4, CS5, CS7, CS10, CS15, CS19, CS20, and CS26 are classified into a similar group, while CS1, CS3, CS6, CS8, CS16, CS17, CS21, CS25 and CS28 are classified into another group. Thus, there is a high possibility that the counterfeit banknote samples that belonged to the two groups were produced by different manufacturers. The fact that c-FACS managed to provide better resolution (identifying three different manufacturers) than that of PCA, it can be construed that the former may have better forensic values.

## Conclusion

The advancement of technology has led to more advanced counterfeiting and fraud of various items and products, including banknotes. Thus, a reliable and effective method is crucial and required, in order to verify the authenticity and to discriminate between genuine and counterfeit products. The chemometrics fuzzy autocatalytic set (c-FACS) is a fuzzy graph method that was introduced for complex data analysis, and has been used in authentication analysis and discrimination of food products. In this paper, the c-FACS is used to authenticate the genuine and counterfeit RM50 banknotes. The results of c-FACS are compared against PCA. The c-FACS outperformed PCA with respect to computing time. On top of that, the distinct and signature patterns of counterfeit RM50 were successfully identified using c-FACS, and thus can be used as signature identification for detecting counterfeit RM50 banknotes. In addition, the c-FACS is able to discriminate the counterfeit samples into three groups, which indicate the possibilities that the samples were produced by three different manufacturers. Thus, the capability of c-FACS in identifying the signature pattern of counterfeit RM50 banknotes and differentiating their sources are shown, and its possibilities for other applications are endless.

## Supplementary Information

Supplementary Information.
