# Supplementary material for: Counterfeit fifty Ringgit Malaysian banknotes authentication using novel graph-based chemometrics method
Source: Sci Rep. 2022 Mar 22;12:4826. doi: 10.1038/s41598-022-08821-w (PMC8941002; doi:10.1038/s41598-022-08821-w)
Supplement: Supplementary file 1 — Supplementary Information. [file 41598_2022_8821_MOESM1_ESM.pdf]

Appendix A

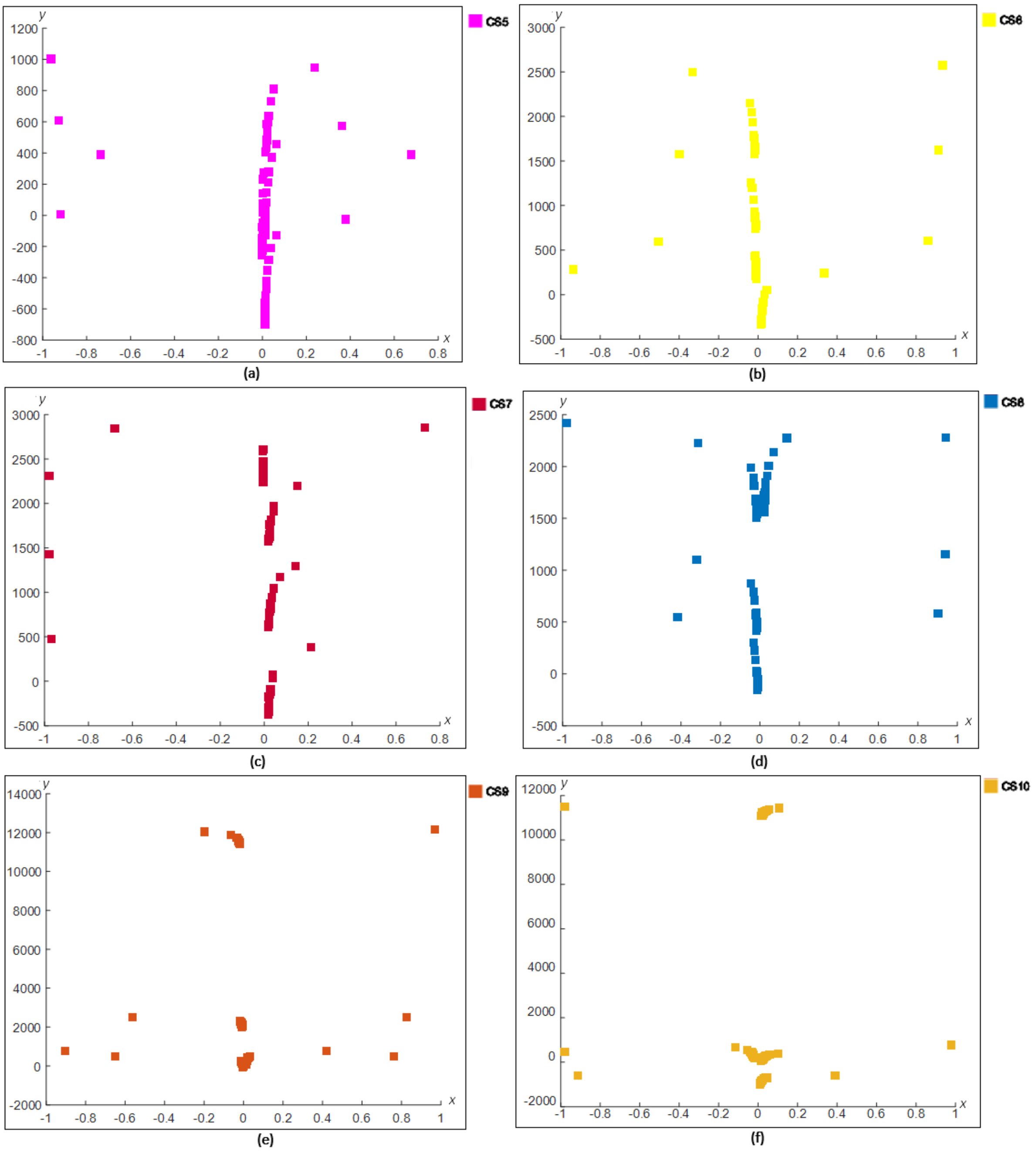

Figure A1. Coordinated FACS of Individual Counterfeit RM50 a) Sample 5 (CS5), b) Sample 6 (CS6), c) Sample 7 (CS7), d) Sample 8 (CS8), e) Sample 9 (CS9) and f) Sample 10 (CS10)

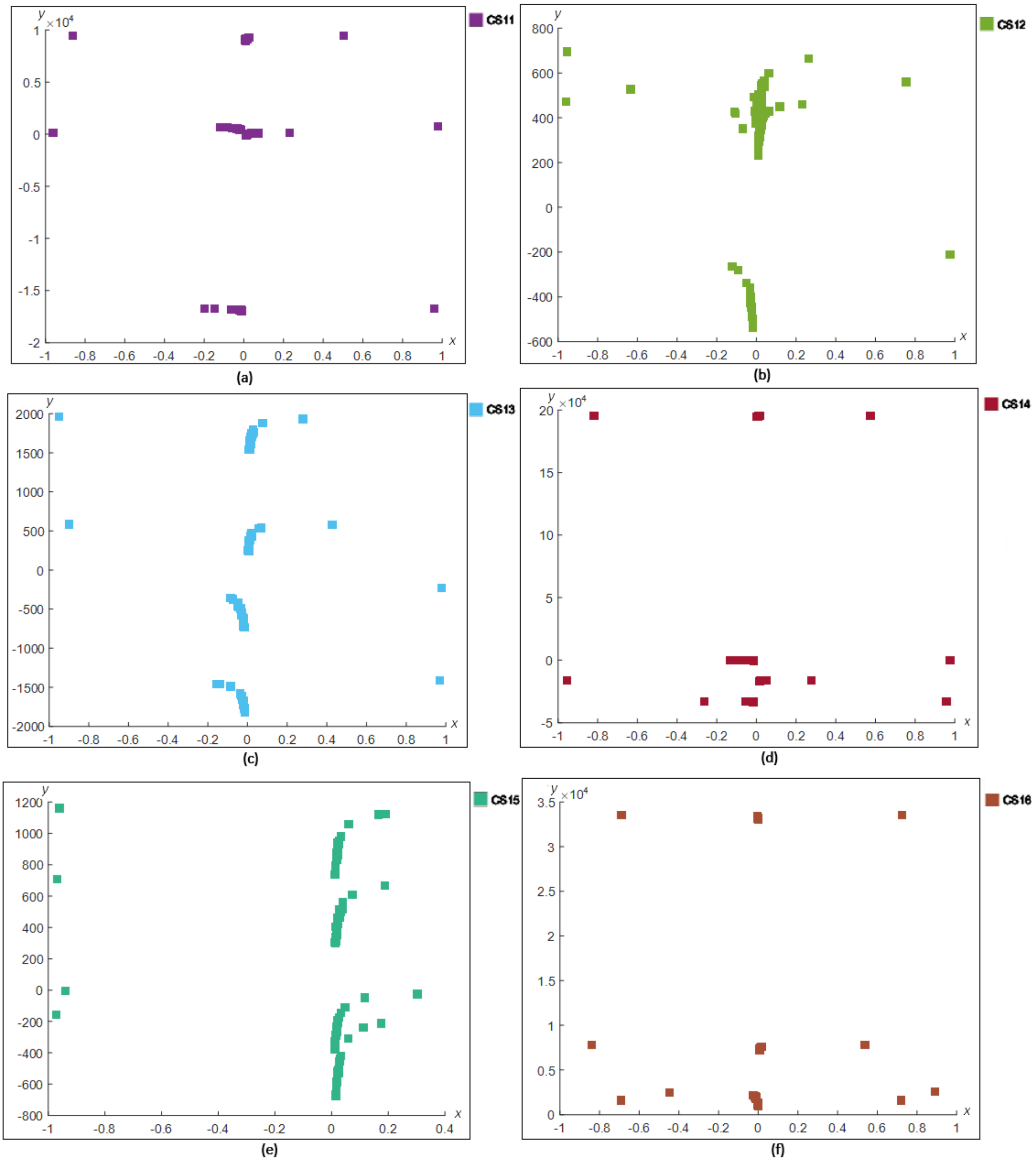

Figure A2. Coordinated FACS of Individual Counterfeit RM50 a) Sample 11 (CS11), b) Sample 12 (CS12), c) Sample 13 (CS13), d) Sample 14 (CS14), e) Sample 15 (CS15) and f) Sample 16 (CS16)

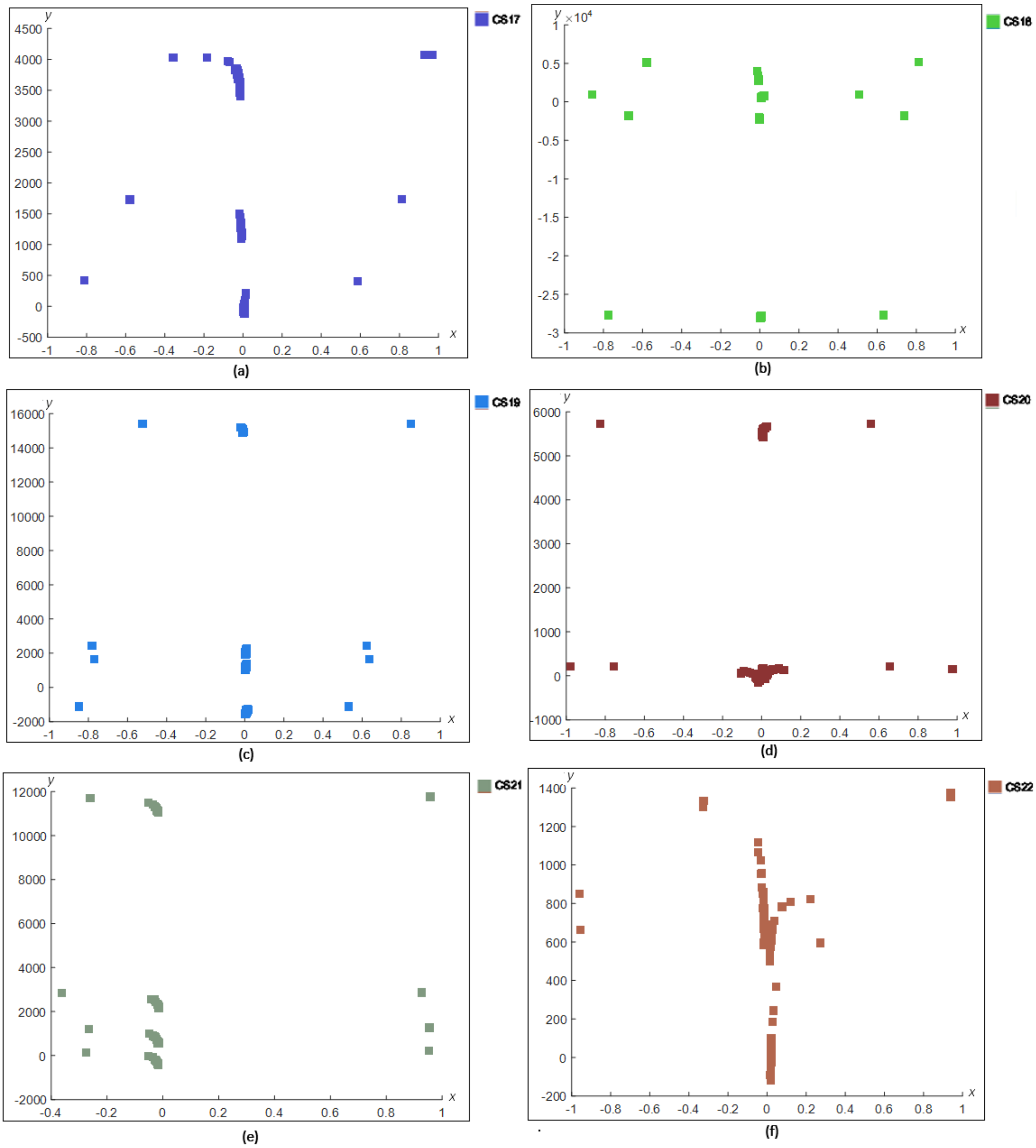

Figure A3. Coordinated FACS of Individual Counterfeit RM50 a) Sample 17 (CS17), b) Sample 18 (CS18), c) Sample 19 (CS19), d) Sample 20 (CS20), e) Sample 21 (CS21) and f) Sample 22 (CS22)

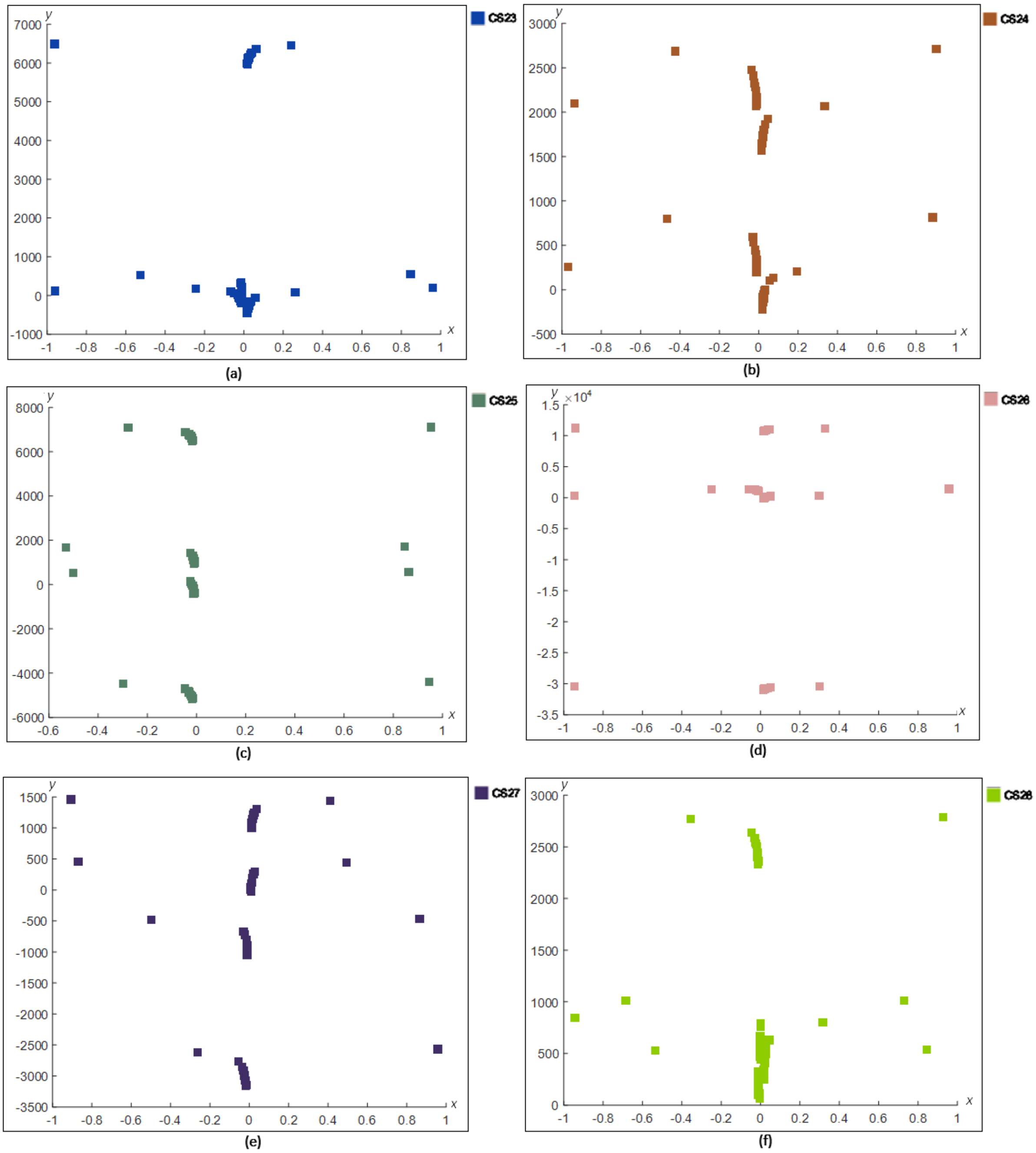

Figure A4. Coordinated FACS of Individual Counterfeit RM50 a) Sample 23 (CS23), b) Sample 24 (CS24), c) Sample 25 (CS25), d) Sample 26 (CS26), e) Sample 27 (CS27) and f) Sample 28 (CS28)
